# Collembola (Hexapoda) as Biological Drivers between Land and Sea

**DOI:** 10.3390/biology10070568

**Published:** 2021-06-22

**Authors:** Izabella Olejniczak, Maria Sterzyńska, Paweł Boniecki, Anita Kaliszewicz, Ninel Panteleeva

**Affiliations:** 1 Institute of Biological Sciences, Cardinal Stefan Wyszynski University in Warsaw, Wóycickiego 1/3, 01-938 Warsaw, Poland; p.boniecki@uksw.edu.pl (P.B.); a.kaliszewicz@uksw.edu.pl (A.K.); 2 Museum and Institute of Zoology, Polish Academy of Sciences, Wilcza 64, 00-679 Warsaw, Poland; majka@miiz.waw.pl; 3 Murmansk Marine Biological Institute, Russian Academy of Sciences, 17 Vladimirskaya Str., 183010 Murmansk, Russia; ninel_panteleeva@mail.ru

**Keywords:** microarthropods, macroalgae, costal tundra

## Abstract

**Simple Summary:**

Collembola also represent one of the most abundant and important taxa of soil invertebrates inhabiting tundra soils. We focused on the structure and distribution patterns of the collembolan communities along the environmental gradient from costal tundra to live macroalgae and aged macroalgae debris deposited across the sub-Arctic coastline. Our results imply that environmental filtering influences collembolan species distributions across the gradient studied and the sorting of species according to their respective functional traits, including dispersal ability. We also suggest that the competition—colonisation trade-off mechanism, which affected the relative importance of competition and environmental filtering, probably determined Collembola community composition over the examined gradient. We believe that our results may become the basis for further study explaining the importance of Collembola as biological drivers of connectivity between land and sea.

**Abstract:**

Macroalgae debris accumulated onshore function as points of interaction between marine and terrestrial ecological systems, but knowledge of the importance of detritivores facilitating the introduction of organic matter via the detritus pathway into neighbouring ecosystems, is still poorly understood. In particular, not much is known about biodiversity patterns and the colonisation of macroalgal debris by terrestrial, detritivorous soil microarthropods in the harsh environmental conditions in the subpolar Arctic region. We hypothesised that (i) soil microarthropods of the coastal tundra, including Collembola, can cross the ecosystem boundary and colonise decaying and freshly exposed macroalgae; and (ii) various inundation regimes by sea water, microhabitat stability and decaying of macroalgae drive distribution patterns of collembolan species. Our results suggest that environmental filtering influences collembolan species’ distributions across the examined gradient and induces sorting of species according to their functional traits, including dispersal ability, resistance to disturbance and environmental tolerance.

## 1. Introduction

Ecological connectivity is regulated by ecosystem boundaries, which function as semi-permeable points of interaction between distinct ecosystems [1]. According to Cadenasso [2], ecosystem boundaries, defined as zones of transition between contrasting systems, can function as ecological filters for habitats, materials or organisms. However, the role of ecosystem boundaries formed along environmental gradients [3] depends on the nature of the neighbouring areas or systems that allow only some fraction of materials, energy or organisms to pass [4]. In our study we treat connectivity between land and sea (land-sea interface) as the introduction of organic matter into the coastal system through soil microarthropods that top-down control the activity of microorganisms in decaying macroalgae.

Coastal transitional zones that occur at the interface of land and sea [5] provide regulation of the fluxes of nutrients, water, particles and organisms to and from the land and the ocean [6] and support a critical habitat for a wide range of both marine and terrestrial biodiversity [7]. Several studies highlight the importance of ecological connectivity and resource transfer from marine to terrestrial ecosystems across ecosystem boundaries for maintaining productivity and diversity at landscape scale [8], including either the distributions of wrack cover in the coastal recipient ecosystem [9], the flow of microbially mediated organic material or both [10].

On the coasts of the polar regions, accumulated onshore macroalgae debris, mainly derived from kelp forests [11], is a material that crosses habitat boundaries and subsidises coastal ecosystems, including residential populations. This material functions as a semi-permeable point of interaction between marine and terrestrial ecological systems and has an important effect on the diversity and distribution pattern of biota [10,12,13], including a considerable bottom-up effect on detritivorous communities [14,15]. Nonetheless, the knowledge of the interconnection and interdependence of marine, coastal and terrestrial ecosystems, including the importance of detritivore communities facilitating the introduction of organic matter into neighbouring ecosystems, remains unclear. In particular, little is known about how deposited onshore macroalgal detritus, which represent cross-boundary subsidies, are either permeable to soil microarthropods, impact their distribution pattern and diversity or both.

Soil microarthropods, by initiating the process of detrital decomposition (shredding, consuming and transforming), accelerate microbial decomposition and nutrient cycling and mediate substantial fluxes between the aboveground and belowground components of terrestrial ecosystems [16]. The decomposition process is particularly important in the Arctic region, where invertebrate communities create relatively simple trophic nets sensitive to any disturbances [17,18]. Among soil microarthropods of the Arctic region, Collembola are the relevant part of the decomposing food web involved in the recycling of organic matter and nutrients in soil [19]. Belowground food web models usually place Collembola as feeding generalists [20]; however, fungi, bacteria and algae may prevail in their diet [19,21]. This triggers top-down control of the decomposer community (i.e., bacteria and fungi) and indirectly may affect soil processes such as organic matter decomposition [19,21] and contribute to soil structure and humus formation [22]. Collembola are also useful models for ecological studies, as their abundance and distribution pattern of species are strongly dependent on individual tolerance limits to environmental conditions [23,24].

Several collembolan species are known to live in either marine wrack material, salt marshes, littoral zones, or a combination [25,26,27]. These terrestrial colonisers of intertidal habitats, resistant to stress and exposed to pronounced environmental fluctuations, are often found in the intertidal environment in very large numbers [28]. Although collembolan species are terrestrial, some of them have adapted to marine habitats where environmental parameters are constantly changing [29]. Collembola also represent one of the most abundant and important taxa of soil invertebrates inhabiting tundra soils [18,30]. Their adaptations to the harsh climatic conditions of polar and subpolar areas [31,32] allow them to survive low winter temperatures [33] and avoid the negative impact of sub-Arctic climate changes induced by drought through either migrating to wetter sites both vertically, horizontally or both [34], through limiting evaporative water loss due to their impermeable cuticles or in combination [35,36]. However, not much is known about either the distribution pattern and permeability, colonisation of the tundra soil microarthropods, including Collembola, to deposited onshore macroalgae debris in such harsh climatic conditions of polar and sub-polar areas, or both.

The current study focuses on the structure and distribution pattern of the collembolan communities along the environmental gradient from costal tundra to living macroalgae and decaying macroalgae debris deposited across the sub-Arctic coastline (Murman coast). There is well-documented coastal habitat connectivity for Collembola [37,38,39]; however, the colonisation patterns of macroalgae by collembolan species has been poorly investigated in subpolar and polar regions [30,40,41]. It should be emphasised that the importance of macroalgae for the diversity and distribution pattern of soil microarthropods, including Collembola, resulting from the interaction between terrestrial and marine ecosystems of the Barents Sea, is not yet known.

To address this knowledge gap, we investigated colonisation patterns of macroalgae by collembolan communities across environmental gradients from coastal tundra to macroalgae (aged macroalgae debris accumulated onshore and living macroalgae freshly exposed by the outflow). We hypothesised that (i) soil microarthropods of the coastal tundra zone, including terrestrial Collembola, can cross the ecosystem boundary and colonise marine macroalgae; and (ii) various inundation regimes by sea water, microhabitat stability and decaying of macroalgae drive distribution patterns of collembolan species.

## 2. Materials and Methods

### 2.1. Study Area and Sampling Processing

**Figure 1:**
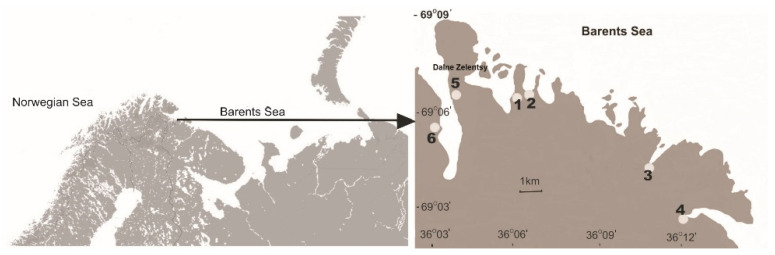
Map of study sites located within the bays at the Barents Sea: 1. Dalne-Zelenetskaya, 2. Plohye Chevry, 3. Medvezhya, 4. Parchniha and 5. and 6. Yarnyshnaya.

The study was conducted on the shoreline of the Murman coast (northwest Russia) of the Barents Sea near the Dalnye Zelentsy settlement (69°7′ N, 36°3′ E) located on the Kola Peninsula (Figure 1). The Barents Sea is a sub-Arctic ecosystem located between 70 and 80° N, connected with the Norwegian Sea to the west and the Arctic Ocean to the north. The salinity of the offshore waters washed onto the Murman coast is about 34% [42] A wide development of the polar front phenomenon and vertical water circulation are the basis of high biological productivity in the sea and high richness of its pelagic and bottom life required for dense growth of kelp forests [43]. They are deposited on the coastal line as spatial subsidies (macroalgae debris) [44].

The northwest Murman coast is characterised by the presence of rocky areas and fjords and is covered with vegetation characteristic of the sub-Arctic tundra [45]. The climate of the Murman coast is rather mild because of the Gulf Stream and is characterised by a humid, cool summer and a relatively mild winter, so the frost-free season lasts about 120 days [45]. The warmest month is July, and the average temperature ranges from 9 to 11 °C; the coldest month is February, which is characterised by an average temperature of about −10 °C [45]. The annual precipitation ranges from 300 to 400 mm [45]. The territory is affected by strong winds and drastically changeable weather [30]. The average temperature, precipitation and wind force in July 2010 and 2013 are given in Table 1.

The study was carried out in the bays located along the Murmansk coast (Figure 1). Dalne-Zelenetskaya Bay (B1) is characterised by a relatively mild onshore slope, with a coastal tundra overgrown mainly with black crowberries, mosses, lichens and grasses, dwarf birch and willows. Plohye Chevry (B2) is stony, with fragments of coastal tundra that function as sea meadows with numerous grasses, sedges and perennials resistant to salinity that have penetrated into the tidal zone and are separated from the tidal zone by a steep fjord. Medvezhya Bay and Parchniha Bay (B3 and B4, respectively) are characterised by a gentle onshore slope, with a coastal tundra covered by mosses, grasses and sedges, respectively, and tundra vegetation at higher elevations not inundated by tidal water that consists of lichens, black crowberries and dwarf birch. Yarnyshnaya Bay, unlike all the previous bays, cuts deeply into the land. The study sites were located on the east and west coasts of the bay, due to the differences in the onshore slope and the vegetation overgrowing them. The eastern side of the bay, with a gentle slope, is covered mainly with a thick layer of black crowberries, lichens, mosses and dwarf birches (B5), while the western side is covered mainly with lichens and mosses with the addition of dwarf birch, often visited by sea birds (B6). Within each bay, sampling was performed across environmental gradients from coastal tundra to decaying macroalgae debris not inundated by tidal water to living macroalgae freshly exposed by outflow.

Sampling was carried out in July of 2010 and 2013 (after the spring tide). On each occasion, the samples were collected from the non-inundated sea water coastal tundra zone (T), then, in the old algae (OA), macroalgae debris zone which had been deposited on the shore at least three weeks earlier, and finally in the fresh algae (FA), which is freshly exposed by outflow (one hour before sampling), living macroalgae zone which had been regularly inundated (twice a day) by sea water. In a given site (B1–B6), 10 samples were taken in each of the study zones (total of 30 samples in each site). The samples were taken every 2 m to a depth of 5 cm using a 10 cm side frame. The depth of the samples was connected to the thickness of the macroalgae, which was about 5 cm. Because study sites involved inundated, living macroalgae, the standard frame method with defined surface area was used, which was suitable for all environments. Collembola from the samples were extracted in Tullgren’s apparatus.

The identification of specimens was based on recent comprehensive manuals [25,26,46,47,48,49,50]. All specimens were deposited at the Cardinal Stefan Wyszynski University in Warsaw, Institute of Biological Sciences (Warsaw, Poland).

### 2.2. Macroalgal Nutrients

Macro- and micronutrient (phosphorous, P; potassium, K; calcium, Ca; magnesium, Mg; manganese, Mn; iron, Fe) content in the living macroalgae freshly exposed by outflow and decaying macroalgae debris was used as a proxy of fertiliser regime (properties) and nutrient supply for microbial growth and bacterial biofilm formation. This content in macroalgal detritus was quantified in 400 mg of algal material digested in 7 mL of concentrated nitric acid and then in an MLS Turbowave microwave liquid digestion system (Anton-Paar Multiwave 3000 microwave unit; Anton-Paar, Graz, Austria), where deionised H_2_O was added to the capacity of 100 cm^3^ and analysed by inductively coupled plasma optical emission spectroscopy (ICP-OES) with an ICPE-9820 simultaneous ICP atomic emission spectrometer (Shimadzu, Kyoto, Japan).

### 2.3. Data Analysis

We used Kruskal–Wallis one-way ANOVA to assess differences in collembolan density, richness and diversity as well as the composition of ecological traits between coastal tundra and decaying and fresh macroalgal detritus patches. The significance of differences was determined with a multiple comparison post hoc test of mean ranks (Dunn’s test) applied after the Kruskal–Wallis ANOVA. The offshore/onshore habitat matrix permeability to Collembola at the sub-Arctic seashore was evaluated by non-metric multidimensional scaling (NMDS). NMDS is a data reduction technique that projects multivariate data along latent axes in a distance measure-based space and preserves the underlying dissimilarity structure between points [51]. The NMDS distance matrix was calculated using the square-root transformed Bray–Curtis distance between cases (samples) [52]. Principal component rotation was employed to maximise the variation of the scores resulting from the NMDS. The NMDS solution was projected in K = 2 dimensions, Kruskal stress algorithm type 2, which uses the sum of squares of the starting differences [52]. To evaluate the goodness of fit for the final NMDS model, we considered stress values of <0.05 as showing an excellent representation of the data: <0.1 good representation, <0.2 acceptable representation and >0.3 unsatisfactory representation [53].

The effect of the land-sea interface on springtail community variation and the distribution pattern was assessed by canonical correspondence analysis (CCA). CCA was employed because, in our case, the value of the gradient calculated for the first axis of detrended correspondence analusis (DCA) was 3.45 SD [54]. The CCA analysis was constrained by factors such as location of site within the bays (B), habitat (H) related to FA, OA and T, and time (T) related to year of study. A partial canonical correspondence analysis (pCCA) was performed to reflect the relative importance of study factors as a group of predictors of Collembola community variation. According to the factors examined, we excluded the possible effect of site (B) in partitioning of the response effect to habitat patch (H) and time (T). The Monte Carlo permutation test was used to quantify the significance of the CCA and pCCA models. In our study, B was the unit of replication (*N* = 6); therefore, for the significance level of the effect of the habitat patch and time, we specified a permutation within blocks defined by the covariate B. Raw and adjusted variation using the number of degrees of freedom was estimated in the CCA and pCCA analyses. A generalised linear model (GLM) was performed to analyse the species-specific distribution of Collembola along the environmental (habitat) gradient examined. A regression model was fitted with a second-order polynomial of the predictor variable and quasi−Poisson distribution for the response data and F-test-based selection. The predictor variable was the first DCA axis fitted with the habitat scores. Species’ response curves were fitted for species that occurred at least in the coastal tundra and macroalgal patches. Among species that met this criterion were *Hypogastrura viatica*, *Tetracanthella arctica*, *Folsomia quadrioculata*, *Entomobrya nivalis*, *Friesea mirabilis*, *Agrenia bidenticulata*, *Pseudisotoma sensibilis* and *Isotoma anglicana*.

Analyses were performed using a data matrix with mean abundance data in which the value in each cell was the mean abundance of species retrieved from 10 soil cores at each plot on each sampling occasion. In the multivariate analysis, time was treated as repeated measures for adjusting *p*-value data. Before calculation, all data were standardised to m^2^ basis, and abundance data for each species were log(x10 + 1) transformed prior to ordination. The level of significance in all analyses was at α = 0.05. Calculations were made with Statistica 10.0 and Canoco 5.0 software packages.

### 2.4. Functional Trait-Based Analysis

The dispersal ability and life form groupings according to morphology-based Gisin’s system [55], modified by Stebajeva [56], were used to evaluate the Collembola community penetration between vegetated low-arctic coastal ecosystems and macroalgal patches across the shoreline of the Barents Sea.

The trait-based structure of Collembola communities was analysed on the basis of the sum of density (m^2^) of species attributed to each trait in each of 10 cores. In this study, we divided Collembola species into epigeic (including species occurring on the ground, vegetation or on the water surface), hemiedaphic and euedaphic life form traits considered as dwellers of litter surface, litter depth and topsoil, respectively, and according to their dispersal ability (fast dispersion, slow dispersion) [57]. The assignment of Collembola species determined during the study to individual life form traits was mainly based on the analysis of various literature resources, including a number of specified synopses and identification keys [25,26,46,47,48,50].

## 3. Results

### 3.1. Macroalgal Fertility Regime

Significant differences in macro- and micronutrient content were found between the FA and OA with respect to K, Ca and Mg (Table 2). OA without wave oscillations (disturbances) had a higher Ca content and a lower Mg and K content.

### 3.2. Changes in Communities of Collembola

A total of 2152 Collembola representing 18 species were identified across all bays on the Murman coast shoreline of the Barents Sea (Table 3). Amongst them, only *Hypogastrura viatica* and *Isotoma anglicana* were recorded in the FA, and *Protaphorura bicampata* were recorded exclusively in OA. Significant variation in density (D), species richness (S) and value of Shannon’s diversity index (H’) of collembolan communities was found amongst coastal tundra and FA and OA (Kruskal–Wallis one way ANOVA:H (2, *N* = 30), *p* < 0.05 in all cases) (Table 3). The multiple comparison post hoc test of mean ranks (Dunn’s test, *p* < 0.05) showed a significantly lower richness, diversity and density of collembolan communities in the living macroalgae exposed by outflow (FA) in comparison to aged macroalgae debris (OA) and coastal tundra (T). Collembolan density had significantly higher mean values in the OA (Table 3).

A significant effect of environmental change across the coastal tundra (T) and old algae (OA) and fresh algae (FA) was found for ecological traits grouped according to life forms, such as mean density of epigeic, hemiedaphic and euedaphic species, and mean density of species with fast and slow dispersal ability (Kruskal–Wallis one way ANOVA:H (2, *N* = 30), *p* < 0.05 in all cases; Table 4). Epigeic and fast-dispersing collembolan species were only able to colonise FA. The mean density of epigeic and fast dispersing species was significantly higher within OA compared to coastal tundra (T) and FA (Dunn’s test, *p* < 0.05). The mean density of hemiedaphic and euedaphic species with slow dispersal ability was significantly lower in OA compared to coastal tundra (T) (Dunn’s test, *p* < 0.05).

**Figure 2:**
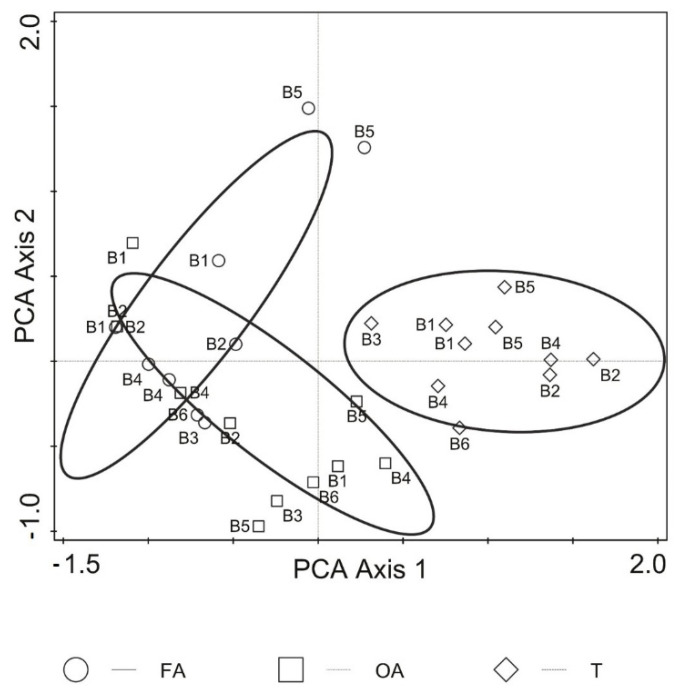
Non-metric multidimensional scaling (NMDS) results of Collembola community distribution pattern amongst study habitats (H) and location (B) of the Barents Sea. FA: alive macroalgae, OA: aged macroalgae, T: coastal tundra; digits 1–6 at variant symbols denote replication of sites (B) across time (2010 and 2013 seasons). NMDS model calculated with Bray–Curtis dissimilarity measure. Axes I and II rotated by applying principal component analysis (PCA) and 37 interactions.

The NMDS results showed dissimilarity between the collembolan communities of coastal tundra and intertidal zones, but revealed proximity between FA and OA within the intertidal zone along the principal component analysis (PCA) rotated axis 1 (Figure 2). The first two axes of NMDS accounted for 92.9% of the total variation in collembolan communities (adjusted explained variation—82.9%, Kruskal stress value = 0.13).

The total CCA model demonstrated that the factors examined accounted for more than 50.4% of the variance in collembolan community composition (Table 5).

**Figure 3:**
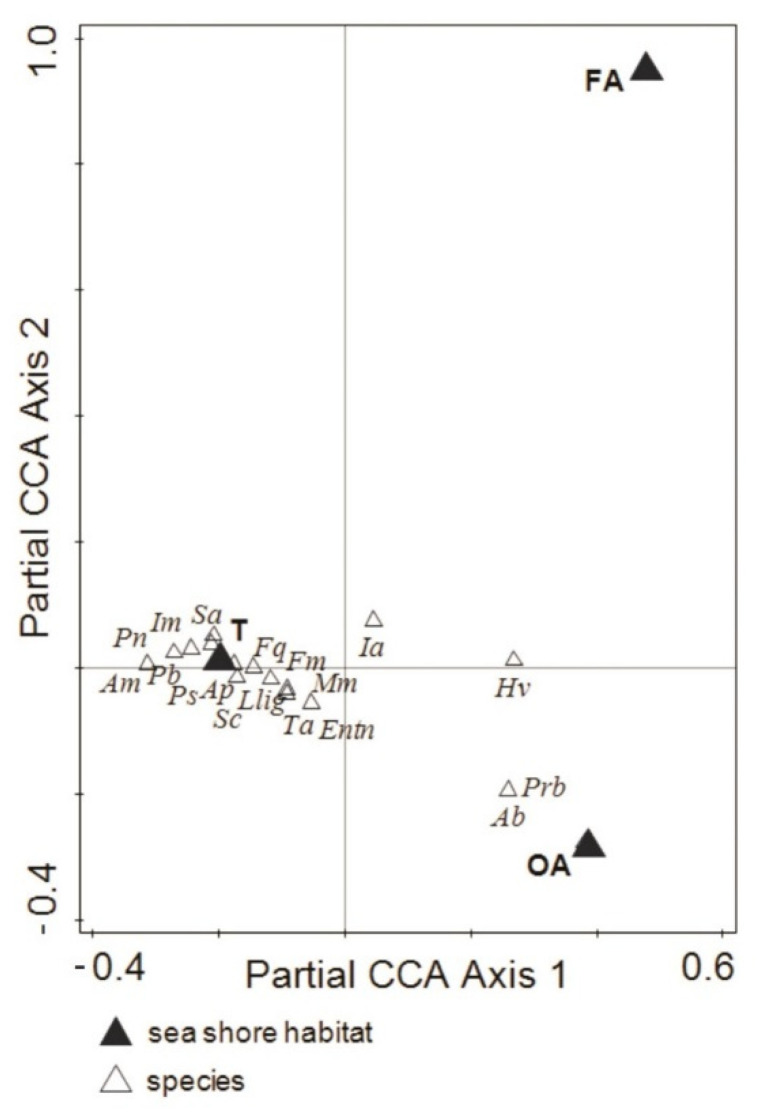
Ordination of the Collembola species in different macroalgal patches and coastal tundra of the Barents Sea. A partial constrained analysis (pCCA) with site (B) and time (Tm) as covariates calculated with log(x10 + 1)-transformed species data; significance level of the effect attributable to covariate bay as block tested by Monte Carlo permutation test under a model with 499 permutations. The full names of the abbreviated species are given in Table 3. T: coastal tundra, OA: old algae (decaying macroalgae debris), FA: fresh algae (living macroalgae).

The pure environmental effect of location (B) and habitat (H) was significant (Monte Carlo permutation test *p* = 0.002) and explained 30.7% (adj. 14.2%) and 30.0% (adj. 24.3%) of the variation, respectively, while variation uniquely attributable to time (Tm), although significant, explained less variation in collembolan communities (10.1%) (adj. 5.8%). The results of pCCA revealed a significant species–environment relationship across the environmental gradient. For instance, *Protaphorura bicampata* and *Agrenia bidenticulata* were associated with OA, while *Isotoma anglicana* and *Hypogastrura viatica* showed a positive correlation with FA (Figure 3).

**Figure 4:**
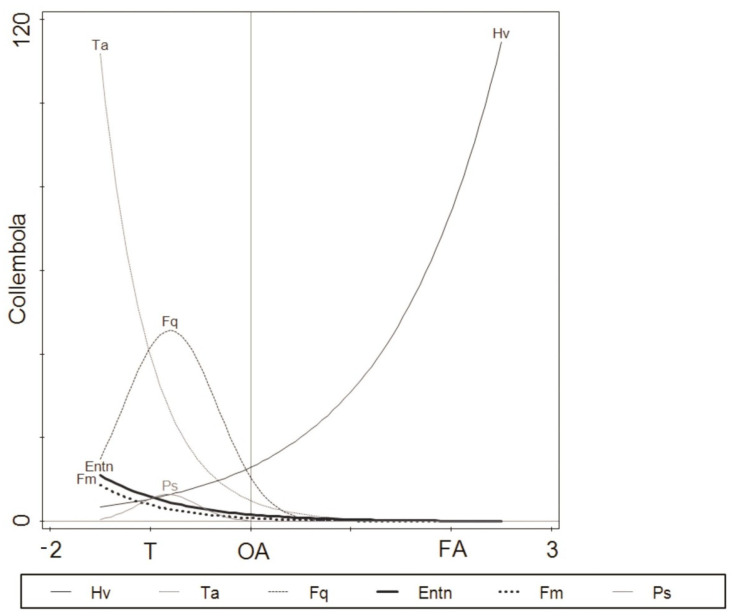
Fitted Collembola species response models, using a generalised linear model (GLM) with a second-order polynomial of the predictor variable (habitat scores). T: coastal tundra, OA: old algae (decaying macroalgae debris), FA: fresh algae (living macroalgae). The full names of the abbreviated species are given in Table 3.

The species response curves fitted with a GLM also indicated differences in species distribution across the examined seashore habitats (Figure 4).

In our case, the total amount of variation was associated with the distribution pattern of *Folsomia quadrioculata* and *Pseudisotoma sensibilis* (79.6% and 85.2%, respectively) fitted with the second-order polynomial predictor. The optimum positions of these two species on the first DCA axis were correlated with coastal tundra along the seashore gradient (habitat gradient) (Figure 4, Table 6). For species such as *Hypogastrura viatica*, *Tetracanthella arctica*, *Entomobrya nivalis* and *Friesea mirabilis*, a linear predictor fitted the species data better, with the lowest explained variation for *Hypogastrura viatica* (18.3%). The pattern in the response curve of *Hypogastrura viatica* was positively correlated with macroalgal habitats (Figure 4, Table 6). No significant responses were shown for *Agrenia bidenticulata* and *Isotoma anglicana*.

## 4. Discussion

### 4.1. Community Structure and Diversity

One of the main goals of our study was to assess whether soil microarthropods, including Collembola representing detritivores, can cross the ecosystem boundary and colonise macroalgae in the subpolar region. To achieve that goal, we examined the distribution pattern of Collembola from the coastal tundra to the macroalgae of the intertidal zone. As expected, our results revealed that macroalgae function as a microhabitat for soil microarthropods. We also clearly demonstrated that terrestrial Collembola can cross the ecosystem boundary between coastal tundra and macroalgae. The apparent differences in community composition and values of richness, diversity and abundance of Collembola across the gradient investigated in our study positively verified our hypothesis that not only the decaying of macroalgae debris but also the stability of microhabitat affect the colonisation pattern.

We noted that inundated by tidal water, FA were colonised only by two epigeic and fast-moving collembolan species: *Hypogastrura viatica* and *Isotoma anglicana.* Both species are considered superhydrophobic [58]. *Hypogastrura viatica* is a cosmopolitan species preferring moist habitats [50], and it is resistant to salinity, adapted to regular flooding [27,29,41] and frequently found along the seacoast [26,29], while *Isotoma anglicana* prefers wet habitats and is commonly found in polar and subpolar areas [25]. The OA, in total, were colonised by a much higher number of epigeic and fast-moving species of Collembola (nine species). Further CCA results revealed that the taxa highly (exclusively) associated with OA were *Protaphorura bicampata*, a species characteristic of wet habitats, such as sea meadows and wrack deposits [25], and *Agrenia bidenticulata*, a species common in damp Arctic tundra, mostly littoral [29] and tolerant to short-term flooding by sea waters [26]. Surprising was the absence of *Archisotoma megalops*, a littoral, fast moving species [30] in macroalgae, especially that was present in the tundra. The absence of this species in the macroalgae patches could be related to either the involved abundance based, not activity-based method or it could be related to its lower density and higher activity in the investigated habitats, or in combination.

Our results also reported a significantly enhanced abundance of collembolan communities in non-inundated OA in comparison with inundated, freshly exposed by outflow FA and coastal tundra sites. These findings suggest that the steep gradient between the terrestrial and marine interface, which induces changes in environmental conditions and environmental stability in the face of disturbances, functions as a filter shaping the communities. Environmental filtering, in the context of species abundance changes in collembolan communities across an environmental gradient, can be explained by habitat preferences and dispersal ability processes [59]. The revealed low permeability of the FA can be related to their extraordinary dynamics associated with inundation by tidal waters. Sea water induces, amongst other things, either osmotic and oxygen stress, changes in salinity, which can limit colonisation/immigration processes and permeability of this harsh and highly dynamic marine macroalgal microbiome to terrestrial Collembola, or in combination [10].

Furthermore, either the small-scale colonisation pattern, penetration of marine subsidies sensu Polis [44] by Collembola across the land-ocean interface in the coastal zone of the subpolar region or both, can also be impacted by biotic interactions, including food requirements and competition for food resources.

Our study highlighted significant differences in sources of nutrients between FA and OA related to the fertiliser regime and microbial concentration. Collembola are generally considered detritivores [36], but their diet also includes fungi and bacteria [19]. The pathway of macroalgal detritus degradation, which can take several weeks [60], facilitates the growth of microorganisms [8] and, in consequence, induces changes in the food resources and food availability for soil microarthropods, including Collembola [41]. Microbial decomposition of macroalgal detritus leads to the release of nutrients and induces changes in trophic interactions that might influence soil biodiversity [61]. Limitations in the availability of food resources as a factor determining the species richness of springtails have been presented in studies of springtail communities in salt marshes [28] and caves [62]. The increase in Collembola abundance in aged macroalgal patches compared with coastal tundra soils, as demonstrated in our study, can also be related to competition for resources and predation. Competition for shared resources and predation are usually amongst the most important factors driving prey community dynamics [63]. Unfortunately, it was impossible to examine resource concentration in the coastal tundra soils and temporal variability in resource availability caused by resource pulses. Therefore, top−down control of collembolan abundance by predation and their competition for resources as a function of resource concentration changes across the gradient investigated were not identified. However, strong competition for resources and extensive niche overlap amongst the dominant arthropod predators in the tundra have been documented [64], while macroalgae, although a rich source of nutrients, unlike the tundra, do not provide many microhabitats and possibilities for a variety of resource use and are characterised by strong competition for resources [29,40].

### 4.2. Functional Traits

Our study also indicated that the community traits of life forms and dispersal ability responded to the steep gradient between terrestrial and marine subsidies. We found higher proportions of collembolan species adapted to the surface way of life (epigeic life forms) and greater dispersal ability in macroalgae in comparison with coastal tundra sites. We also noted that the decaying macroalgal patches, unlike the fresh patches, promoted coexistence of a higher number of Collembola species adapted to the surface way of life and demonstrated greater colonising ability. This result suggests that competition—colonisation trade-off mechanism—can probably determine community composition over the examined gradient [65]. The apparent differences in total abundance of epigeic and fast dispersed species between FA and OA, being an intermediate stage of the gradient between coastal tundra and FA, suggest that disturbance intensity can shape the species trait patterning. This finding confirms community functional responses to disturbances [66] and the relationship between ecological preferences or tolerances of the collembolan species and species trait patterning [59,67].

### 4.3. Ecological Species Filtering

The NMDS results, however, showed weak penetration of terrestrial springtails into the marine subsidy (Figure 2). It is known that ecological constraints and spatial distance between patches can influence the assembly and composition of communities [68]. The results of our NMDS model suggest that, in such a highly dynamic and unstable tidal system, it is the ecological constraints rather than spatial distance between patches that probably play a pivotal role in the assembly of collembolan communities. The distances between patches across the study transects were small (about 1 m) and probably did not limit migration of the Collembola. According to some studies, the dispersal pathway of Collembola could be active [69,70,71], e.g., by flooding water or strong wind that dominate in the Arctic coast that we studied. Therefore, the finding of high dissimilarity amongst collembolan communities in our study indicated that ecological constraints, such as either biotic interactions (competition) [72,73], abiotic conditions (e.g., either salinity, nutrient contents or both), habitat instability or in combination, had a key effect on their dispersal patterning.

Nonetheless, it is important to note that local differences between the bays related to coastline topography, habitat patch and seasonal variability were significant drivers of variability in collembolan community composition. Variance partitioning in CCA models showed that location (B) and habitat patch (H) had the greatest unique effect on the variation of collembolan community and explained similar proportions of their variation, while time, although significant, explained less variation (Table 5). This finding points to the role of environmental conditions acting as filters for local species sorting [74]. Soil microarthropod communities can be sorted by environmental filtering [59,68], including species tolerance to inundation and salinity [75].

Further GLM models (Figure 4) confirm that environmental filtering selects species with particular traits. The results of our study showed the disappearance of species connected to the Arctic tundra, such as *Tetracanthella arctica* [30,48], along the seashore gradient (Figure 4) and increased abundance of surface water-dwelling species resistant to salinity, such as *Hypogastrura viatica*, in macroalgal patches. This finding confirms Widenfalk and others’ concept that the degree of environmental filtering by local communities is dependent on the degree of environmental disturbance [73]. Our results show that, although most of the terrestrial species we found in coastal tundra were characterised by a wide range of distribution, only those resistant to flooding, dryness or salinity were able to penetrate from the coastal tundra to macroalgal beds of the intertidal zone. The observed disappearance of species associated with the Arctic tundra, such as *Tetracanthella arctica* [30,48], along the seashore gradient (Figure 4) can be related to habitat preferences and disturbance tolerance. Species with a wide tolerance range of environmental conditions are able to colonise variable environments [27,71], while those revealing a lack of tolerance to new environmental conditions disappear [71]. The replacement of *Hypogastrura viatica* by *Folsomia quadrioculata* in decaying macroalgae (Figure 4) in our study indicates that, besides habitat condition changes (dryness and salinity) that can control the distribution pattern of collembolan species, the competition for food could be a predominant factor. According to some studies, macroalgal patches are decomposed mainly by bacteria [76], which are sources of food for Collembola, including *Folsomia quadrioculata* and *Hypogastrura viatica* [41]. The importance of competition (competitive exclusion) on the collembolan community composition, documented in many studies [72,73], was a predominant factor in the OA of the Kola Bay [40].

## 5. Conclusions

As expected, Collembola can cross the boundary between coastal tundra and macroalgae on the seashore. Nonetheless, it is important to indicate that OA were more permeable to coastal tundra springtails than freshly exposed by outflow FA. These results imply that environmental filtering influences collembolan species distributions across the gradient studied and the sorting of species according to their respective functional traits, including dispersal ability. Our findings also suggest that the competition—colonisation trade-off mechanism, which affected the relative importance of competition and environmental filtering—probably determined Collembola community composition over the examined gradient (dynamics of environmental changes).

## Figures and Tables

**Table 1:** Monthly average temperatures (°C), precipitation (in mm H_2_O) and wind force (in m/s) from the weather station at Teriberka (69°1′ N, 35°1′ E) located on the Barents Sea in July 2010 and 2013. The average temperature, precipitation and wind force in July 2010 and 2013 are given according to the weather station in Teriberka (69°1′ N, 35°1′ E) located on shore of the Barents Sea.

Year	Temperature in °C	Precipitation in mm H_2_O	Wind Force in m/s
2010	12.9	63.9	6.3
2013	14.2	55.3	6.1

**Table 2:** Effect of wave exposure and macroalgal age on the macro- and micronutrient content. Values presented in the table are means across replicates of each algal sample (*n* = 4) with standard deviation (SD). OA: old algae (decaying macroalgae debris), FA: fresh algae (living macroalgae).

Nutrient	OA	FA
TP (mg/L)	1.24 ± 0.46	1.03 ± 0.24
K^+^ (mg/L) *	1.65 ± 0.26	6.43 ± 0.61
Ca^2+^ (mg/L) *	46.75 ± 13.28	22.25 ± 2.06
Mg^2+^ (mg/L) *	18.25 ± 2.06	15.50 ± 1.29
TFe (µm/L)	0.69 ± 0.53	0.23 ± 0.06

* Significant differences at 0.05 probability level tested by Mann–Whitney *U* test.

**Table 3:** Species composition of Collembola communities in the coastal and intertidal zone of the Barents Sea, code of the abbreviated species and classification of the collembolan species functional traits: life forms (Ep: epigeic, He: hemiedaphic, Eu: euedaphic) and dispersal ability (slow, fast). T: coastal tundra, O: old algae (decaying macroalgae debris), FA: fresh algae (living macroalgae), D: density in 10^3^ ind·m^−2^ ± standard deviation.

NO	Collembola	Code	Sea Shore Habitat			Functional Traits	
			T	OA	FA	Life-Form	Dispersal Ability
	Hypogastruridae						
1	*Hypogastrura viatica* (Tullberg, 1872)	Hv	+	+	+	Ep	Fast
	Neanuridae						
2	*Friesea mirabilis* (Tullberg, 1871)	Fm	+	+	−	He	Slow
3	*Anurida papillosa* (Axelson, 1902)	Ap	+	−	−	He	Slow
	Onychiuridae						
4	*Protaphorura bicampata* (Gisin, 1956)	Prb	−	+	−	Eu	Slow
	Tullbergiidae						
5	*Mesaphorura macrochaeta* Rusek, 1976	Mm	+	−	−	Eu	Slow
	Isotomidae						
6	*Tetracanthella arctica* Cassagna, 1959	Ta	+	+	−	Ep	Fast
7	*Pseudanurophorus binoculatus* Kseneman, 1934	Pb	+	−	−	Eu	Slow
8	*Folsomia quadrioculata* (Tullberg, 1871)	Fq	+	+	−	He	Slow
9	*Isotomiella minor* (Schäffer, 1896)	Im	+	−	−	Eu	Slow
10	*Archisotoma megalops* (Bangall, 1939)	Am	+	−	−	Ep	Fast
11	*Agrenia bidenticulata* (Tullberg, 1877)	Ab	+	+	−	Ep	Slow
12	*Pseudisotoma sensibilis* (Tullberg, 1876)	Ps	+	+	−	He	Slow
13	*Parisotoma notabilis* (Schäffer, 1896)	Pn	+	−	−	He	Slow
14	*Isotoma anglicana* Lubbock, 1862	Ia	+	+	+	Ep	Fast
	Entomobryidae						
15	*Entomobrya nivalis* (Linnaeus, 1758)	Entn	+	+	−	Ep	Fast
16	*Lepidocyrtus lignorum* (Fabricius, 1775)	Llig	+	−	−	Ep	Fast
	Katiannidae						
17	*Sminthurinus aureus* (Lubbock, 1862)	Sa	+	−	−	Ep	Fast
18	*Sminthurinus concolor* (Meinert, 1896)	Sc	+	−	−	Ep	Fast
	Total number of species		17	9	2		
	Density (D)		8.65 ± 6.48 a	12.54 ± 17.53 b	0.32 ± 0.25 c		
	Species richness (S)		7.20 ± 1.40 a	2.40.0 ± 1.26 b	1.22 ± 0.44 b		
	Shannon’s index H’		1.39 ± 0.23 a	0.39 ± 0.43 b	0.13 ± 0.27 b		

Bars sharing the same letter are not significantly different (*p* < 0.05).

**Table 4:** Functional trait values in Collembola communities across seashore habitats of the Barents Sea tested by Kruskal–Wallis one-way ANOVA test. Values are means ± standard deviation (SD) across replicates (*N* = 6). Significant differences are marked by different letters in rows; statistical significance tested by a multiple comparison post hoc test of mean ranks (Dunn’s test) applied after Kruskal–Wallis ANOVA with *p* < 0.05. T: coastal tundra, O: old algae (decaying macroalgae debris), FA: fresh algae (alive macroalgae).

Functional Traits	T	OA	FA
Life form:			
Epigeic	3.89 ± 3.75 a	12.43 ± 17.58 b	0.29 ± 0.26 c
Hemiedaphic	4.54 ± 3.42 a	0.10 ± 0.16 b	0
Euedaphic	0.22 ± 0.34 a	0.01 ± 0.03 a	0
Dispersal ability:			
Fast	3.89 ± 3.75 a	12.43 ± 17.58 b	0.29 ± 0.26 c
Slow	4.24 ± 2.47 a	0.22 ± 0.25 b	0

Bars sharing the same letter are not significantly different (*p* < 0.05).

**Table 5:** Effect of site (B), habitat (H) and time (Tm) on variation of Collembola communities across coastal and intertidal habitats indicated by canonical correspondence analysis (CCA) and partial canonical correspondence analysis (pCCA). Pseudo-F: F-statistic, *p*: significance level tested by Monte Carlo permutation test, CCA model calculated with log(x + 1)-transformed data.

Explanatory Variables	Covariates	Covariate Define Blocks	Total Explained Variance (%)	Partial Variance (%)	Adjusted Explained Variance (%)	Pseudo-F	*p*-Value
B, H, Tm			50.4		31.4	2.7	**0.002**
H	B, Tm	B		30.0	24.3	4.5	**0.002**
Tm	B, H	B		10.1	5.8	2.4	**0.016**
B	H, Tm			30.7	14.2	1.9	**0.012**

Significant *p*-values are indicated in bold. Variation account using the adjusted R^2^ approach.

**Table 6:** GLM regression models for selected Collembola species responses to the seashore habitat gradient. Model based on F-test selection. R^2^: coefficient of determination, F: value of the F-ratio statistic, *p*: significance level of the model, O: parameter of species optimum, T: parameter of species tolerance. The full names of the abbreviated species are given in Table 3.

Explanatory Variable	Selected Model	Null Deviance	Deviance under Model	R^2^ (%)	F	*p*	O	T
Hv	linear	4288.0	3502.5	18.3	4.3	0.04737	NA	NA
Ta	linear	879.4	327.6	62.7	43.6	<0.00001	NA	NA
Fq	quadratic	1020.0	208.28	79.6	53.9	<0.00001	−0.800	0.467
Entn	linear	129.1	59.2	54.1	30.0	<0.00001	NA	NA
Fm	linear	121.8	71.1	41.6	13.7	0.00093	NA	NA
Ps	quadratic	126.5	18.8	85.2	50.8	<0.00001	−0.824	0.304

## Data Availability

Not applicable.

## Abbreviations

FA	fresh algae
OA	old algae
T	coastal tundra
B	site within the bays
H	habitat
Tm	time
EP	epigeic life form
He	hemiedaphic life form
Eu	euedaphic life form
pCCA	partial canonical correspondence analysis
CCA	canonical correspondence analysis
DCA	Detrended correspondence analysis
PCA	principal component analysis
GLM	generalised linear model
NMDS	Non-metric multidimensional scaling
D	density of collembolan communities (10^3^ ind·m^−2^)
S	species richness of collembolan communities
H’	Shannon’s diversity of collembolan communities
Hv	*Hypogastrura viatica* (Tullberg, 1872)
Fm	*Friesea mirabilis* (Tullberg, 1871)
Ta	*Tetracanthella arctica* Cassagna, 1959
Fq	*Folsomia quadrioculata* (Tullberg, 1871)
Ps	*Pseudisotoma sensibilis* (Tullberg, 1876)
Entm	*Entomobrya nivalis* (Linnaeus, 1758)
